# Probability-Based Forwarding Scheme with Boundary Optimization for C-V2X Multi-Hop Communication

**DOI:** 10.3390/s26010350

**Published:** 2026-01-05

**Authors:** Zhonghui Pei, Long Xie, Jingbin Lu, Liyuan Zheng, Huiheng Liu

**Affiliations:** 1 School of Computer Science and Engineering, Wuhan Institute of Technology, Wuhan 430205, China; peizhonghui@wit.edu.cn (Z.P.); xielong@stu.wit.edu.cn (L.X.); lujingbin@stu.wit.edu.cn (J.L.); zhengliyuan@stu.wit.edu.cn (L.Z.); 2 School of Physics and Electronic Engineering, Hubei University of Arts and Science, Xiangyang 441053, China

**Keywords:** internet of vehicles, multi-hop broadcast, probability-based forwarding, forwarding redundancy

## Abstract

The Internet of Vehicles (IoV) can transmit the status information of vehicles and roads through single-hop or multi-hop broadcast communication, which is a key technology for building intelligent transportation systems and enhancing road safety. However, in dense traffic environments, broadcasting Emergency messages via vehicles can easily trigger massive forwarding redundancy, leading to channel resource selection conflicts between vehicles and affecting the reliability of inter-vehicle communication. This paper analyzes the forwarding near the single-hop transmission radius boundary of the sending node in a probability-based inter-vehicle multi-hop forwarding scheme, pointing out the existence of the boundary forwarding redundancy problem. To address this problem, this paper proposes two probability-based schemes with boundary optimization: (1) By optimizing the forwarding probability distribution outside the transmission radius boundary of the sending node, the forwarding nodes outside the boundary can be effectively utilized while effectively reducing the forwarding redundancy they bring. (2) Additional forwarding backoff timers are allocated to nodes outside the transmission radius boundary of the sending node based on the distance to further reduce the forwarding redundancy outside the boundary. Experimental results show that, compared with the reference schemes without boundary forwarding probability optimization, the proposed schemes significantly reduce forwarding redundancy of Emergency messages while maintaining good single-hop and multi-hop transmission performance. When the reference transmission radius is 300 m and the vehicle density is 0.18 veh/m, compared with the probability-based forwarding scheme without boundary optimization, the proposed schemes (1) and (2) improve the single-hop packet delivery ratio by an average of about 5.41% and 11.83% and reduce the multi-hop forwarding ratio by about 18.07% and 36.07%, respectively.

## 1. Introduction

The Internet of Vehicles (IoV), as an important component of Intelligent Transportation Systems (ITSs), consists of mobile vehicles and roadside units equipped with sensing, computing, storage, and wireless communication capabilities. The IoV enables Vehicle-to-Pedestrian (V2P), Vehicle-to-Vehicle (V2V), and Vehicle-to-Infrastructure (V2I) communication. IoV technology aims to enable vehicles to perceive their own driving status and surrounding traffic conditions in real time, facilitating large-scale collaborative sharing of traffic information [1,2].

The core mission of the IoV is to enable fast and reliable transmission of security messages, which include Beacon messages and Emergency messages [3]. Beacon messages are periodically broadcast by vehicles to share driving status information (such as GPS location, driving direction, speed, acceleration/deceleration, lane changes, and overtaking maneuvers) with neighbor vehicles. Beacon messages can be transmitted with a single-hop broadcast, while Emergency messages are usually triggered by events such as traffic accidents or dangerous road conditions [4,5]. Due to the limited range of single-hop communication between vehicles, inter-vehicle multi-hop broadcast technology is often necessary to rapidly notify groups of vehicles several kilometers behind the accident vehicle, which is particularly crucial on highways where base-station signals may not provide complete coverage [6]. Multi-hop broadcast refers to a routing protocol where a single sending node transmits messages to multiple surrounding nodes by multi-hop forwarding, serving as a common communication method for transmitting Emergency messages in IoV [7].

The simplest multi-hop broadcast protocol is the Flooding broadcast protocol, where each candidate node that receives a message will forward it by broadcasting until it reaches the destination node. However, in traffic environments with high vehicle density, Flooding can easily trigger broadcast storms [8]. Based on this foundation, numerous researchers have proposed various optimized multi-hop broadcast protocols aimed at effectively controlling broadcast storms while minimizing transmission delays, thereby improving message delivery reliability. For example, the delay-based multi-hop broadcast protocols allow candidate neighbor nodes to adjust their forwarding wait times [9]. Typically, the waiting time of a node is inversely proportional to its distance from the sender. When the waiting time ends, if the current node has not received the next hop for-warding for the same message, it will forward the message; otherwise, the current forwarding attempt will be canceled. However, during the process of multi-hop broadcasting of Emergency messages, the cumulative waiting time per hop may result in excessively long end-to-end propagation delays.

Probability-based multi-hop broadcast protocols typically assign forwarding probabilities to nodes based on parameters such as distance, vehicle density and transmission radius, ensuring the reliability of multi-hop transmission of a message while controlling the number of forwarding nodes [10]. For instance, in the Weighted Probability-Based Broadcasting (WPB) scheme described in [11], the receiving node calculates the forwarding probability based on the ratio of the Euclidean distance *d* to the sending node and the transmission radius *R* to determine whether to forward the message. However, in the actual environment of wireless communication, the effective single-hop transmission radius of each vehicle node may exceed the reference value *R* due to factors such as transmission power, physical layer channel conditions, and modulation schemes. This results in a forwarding probability of 1 for nodes outside the boundary R that successfully receive the message. Therefore, the probability-based forwarding mechanism similar to WPB may easily increase forwarding redundancy of nodes outside of the boundary *R*, especially in road scenarios with high vehicle density, which leads to an increased probability of transmission collisions.

To reduce forwarding redundancy beyond boundary *R* and enhance the reliability of multi-hop transmission for Emergency messages, it is necessary to optimize the forwarding mechanism outside of boundary *R* in the probabilistic forwarding schemes. This paper proposes two optimization approaches to address the aforementioned boundary redundant forwarding issue:

(1) Probabilistic Forwarding Scheme with Boundary Optimization: When the transmission range of the Emergency message exceeds *R*, the distance-dependent forwarding probability is recalculated using *R* as the boundary. By replacing the distance *d*(*x*) between the current node and the sending node with the value of *d*(*x*) modulo *R*, the forwarding probability distribution within and outside of the reference transmission radius *R* becomes consistent. This reduces forwarding redundancy outside of boundary *R* and lowers the collision probability of Emergency messages on the basis of the probabilistic scheme.
(2) Probabilistic Forwarding Scheme with Joint Optimization of Boundary and Delay: For nodes outside of boundary *R*, in addition to calculating distance-dependent forwarding probability, the backoff timer mechanism is introduced for nodes beyond boundary *R*. Upon timer expiration, if no subsequent hop forwarding for the same Emergency message has been received, the current forwarding is executed; otherwise, forwarding is canceled. The backoff timer serves to further reduce forwarding redundancy outside of the boundary.

The remainder of this paper is summarized as follows: Section 2 discusses research progress on multi-hop broadcasting protocols for IoV; Section 3 analyzes the forwarding redundancy problem of nodes outside the boundary and the collision probability model of Emergency messages; Section 4 elaborates on the design principles and implementation process of the proposed schemes. Section 5 compares the performance of the proposed schemes with reference schemes through simulation experiments, focusing on analyzing the transmission performance of Beacon messages and Emergency messages across different schemes. Section 6 concludes the entire work.

## 2. Related Works

To address the inefficiency of Flooding broadcast, researchers have continuously proposed various optimized multi-hop broadcast protocols for IoV. These protocols primarily include delay-based forwarding, location-based forwarding, and probability-based forwarding.

### 2.1. Delay-Based Forwarding

Briesemeister et al. were the first to introduce a delayed forwarding mechanism into multi-hop broadcast protocols [12]. The waiting time of the candidate forwarding nodes they proposed is inversely proportional to the distance from them to the sending node (the node that first initiates the transmission of the Emergency message). The farther the distance, the shorter the waiting time. Subsequent researchers further proposed some algorithms based on [12], for example, ref. [13] proposes a Time-barrier Based Emergency Message Dissemination (TB-EMD) scheme, which similarly assigns shorter waiting delays to more distant vehicles to grant them higher priority of forwarding messages. Differently from the above research, ref. [14] proposes a Density-aware Emergency Message Extension Protocol (DEEP), which dynamically partitions the communication range of the accident vehicle into several logical blocks and allocate a waiting timer to each block. The block further from the accident vehicle has a shorter waiting timer. The following vehicles perceive the block they are in through their own positioning system, and the vehicle that finishes its waiting timer first has priority to forward the message. The Contention Based Forwarding (CBF) scheme in [15] not only assigns waiting times to candidate nodes according to distance, but also dynamically adjusts the contention window based on forwarding priority and vehicle density to reduce packet collisions.

### 2.2. Location-Based Forwarding

Location-based forwarding refers to a multi-hop routing method that makes each message forwarding gradually move closer to the target node based on the positional information of both the destination nodes and the neighbor nodes. The Distributed Position-based Protocol for Emergency messages Broadcasting (DP-EMB) proposed in [16] combines the real-time positions and velocities of sending and receiving vehicles. It assigns shorter forwarding wait times to vehicles that are far from the sending node, accelerating the multi-hop propagation of emergency messages through a geographical priority mechanism. In addition to position, subsequent researchers integrated more metrics into their algorithms to improve the selection mechanism of forwarding node. For instance, ref. [17] proposes a Position-based Reliable Emergency Message Routing (REMR) scheme, the core of which is to select forwarding nodes by utilizing information such as the speeds, movement angles and positions of neighboring nodes. The REMR relies on the timeliness and accuracy of node location information. If the GPS signal is weak (e.g., in a tunnel or due to tall obstacles) or the location information is delayed, it may lead to routing errors, thereby reducing the reliability of message transmission. Ref. [18] also assigns higher forwarding priority to neighbors that are farther away, but it uses active RFID tags deployed on the road instead of GPS for vehicle positioning to improve the accuracy and reliability of forwarding node selection. However, RFID tags increase deployment costs and are not conducive to large-scale deployment.

### 2.3. Probability-Based Forwarding

Probability-based forwarding schemes have attracted much attention from scholars because they do not require additional forwarding timers. Wisitpongphan et al. [11] proposes a Weighted P-persistence Broadcasting (WPB) scheme, where the candidate node calculates probability *P* according to distance from the sending node and the transmission radius *R* after receiving an Emergency message. However, the forwarding probabilities of all nodes that are farther away than *R*/2 from the sending node exceed 50%. Consequently, in road scenarios with high vehicle density, this approach is prone to generating excessive forwarding redundancy and may even trigger broadcast storms. In [19], the authors proposed an Nth-Powered P-persistent Broadcast (NPPB) protocol based on [11]. This protocol also assigns higher forwarding probabilities to nodes farther away. However, if there are insufficient forwarding nodes near the transmission boundary *R*, the probability of successful message delivery will decrease.

To optimize the probability calculation method, some researchers integrated multiple parameters to construct the forwarding probability models. For example, The Probability-based Multi-metric Routing Protocol (ProMRP) calculates the weighted probabilities of neighbors based on parameters such as distance, vehicle density, and bandwidth [20]. ProMRP prioritizes selecting the candidate node with the highest weighted probability in the neighbor table as the forwarding node. Similarly, the scheme in [21] calculates weighted probability based on metrics such as the movement direction of neighbor nodes, channel fading, and link connectivity. It also prioritizes the node with the highest weight as the forwarding node. Differently from [20], if the message fails to be received by nodes of next hop, the node has the second weighted probability will continue to perform message forwarding. Hamdi et al. [22] proposed a multi-hop broadcast protocol based on clustering and probabilistic forwarding, which dynamically adjusts the forwarding probability according to the number of times a node receives a message, where the reception number of messages is inversely proportional to the forwarding probability to reduce the forwarding redundancy of messages. However, if a node is near the communication range boundary of the forwarding node at the previous hop but receives a large number of messages, it may have a very low forwarding probability. The CBR-based Probabilistic Forwarding Protocol (CPF) proposed in [23] utilizes the distance *d* between the receiver and sender along with the Channel Busy Ratio (CBR) to calculate forwarding probability of a candidate node, effectively controlling the number of forwarding nodes within the boundary *R*. According to above literature analysis, probability-based forwarding schemes predominantly rely on reference transmission radius *R* and distance calculations to determine forwarding probabilities. When the actual transmission distance of Emergency messages exceeds the reference value *R*, nodes outside *R* perform forwarding with probability 1. This may easily cause boundary redundant forwarding issues in road scenarios with high vehicle density.

In summary, different forwarding schemes typically have their applicable propagation scenarios. Delay-based forwarding schemes can effectively control broadcast storms but introduce additional forwarding delays, impacting the timeliness of latency-sensitive security messages. Probability-based forwarding schemes can control the number of forwarding nodes, particularly in scenarios with high vehicle density. However, when commonly relying on the reference transmission radius as the basis for probability calculation, it tends to cause forwarding redundancy at the boundary position of the reference transmission radius of the sending node.

## 3. Theoretical Analysis

### 3.1. C-V2X Mode 4 and Analysis of Resource Selection Conflicts

Cellular Vehicle-to-Everything (C-V2X) Mode 4, as a key technology for multi-hop communication scenarios in IoV, employs a distributed resource allocation architecture to enable low-latency V2V communication without the assistance of the cellular infrastructure [24]. To accommodate the transmission requirements of periodic V2X messages such as Beacon messages, C-V2X Mode 4 employs a sensing-based Semi-Persistent Scheduling (SPS) scheme. Vehicles can autonomously select channel resources for message transmission and periodically occupy a specific channel for transmission [25].

However, In V2X communication systems based on C-V2X Mode 4, Emergency messages are typically triggered by specific events, whose packet sizes are typically larger than Beacon messages and need lower transmission delay requirements [26]. Channel resources reserved for Beacon messages often fail to meet the size and delay constraints of Emergency messages. Consequently, the transmission of Emergency messages will trigger the vehicle to execute a resource reselection process.

**Figure 1:**
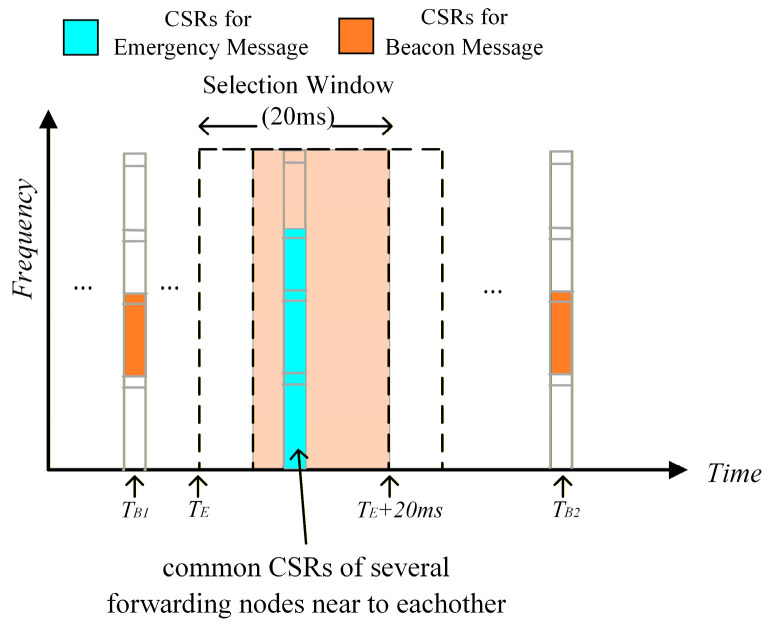
Resource selection windows overlap among multiple forwarding nodes.

Assume that the transmission of a Beacon message occupies one subchannel with a transmission interval and delay requirement of 100 ms, while the transmission of an Emergency message occupies three subchannels with a transmission delay requirement of 20 ms. As shown in Figure 1, when a vehicle *V_A_* experiences an emergency event, it must rapidly broadcast an Emergency message outward to alert other vehicles to take evasive action. Assume that a vehicle *V_B_*, which is near the transmission radius boundary of vehicle *V_A_*, receives the next-hop forwarding of the Emergency message from *V_A_*. Since the channel resources currently reserved by *V_B_* for Beacon messages in the *T_B1_* subframe cannot meet the transmission delay and packet size requirements of the Emergency message, *V_B_* will perform resource reselection. Assume that *V_B_* performs resource reselection at *T_E_* and selects a Candidate Single-subframe Resources (CSR) within [*T_E_*, *T_E_* + 20 ms] for transmitting the Emergency message. If multiple vehicles surrounding *V_B_* also receive the next-hop forwarding of this Emergency message, since these vehicles and *V_B_* nearly simultaneously receive the Emergency message and execute resource selection, they have a resource selection window with significant overlap. This increases the probability of resource selection conflict between *V_B_* and its surrounding vehicles, where the resource selection conflict refers to the probability that multiple vehicle nodes select the same CSR to execute message transmission.

### 3.2. Analysis of Boundary Redundancy Forwarding in Probabilistic Forwarding Schemes

Due to the transmission distance limitations of a single vehicle, Emergency messages that need to be transmitted to more distant nodes in a short period of time usually require multi-hop transmission. Multi-hop communication between vehicles allows vehicles to not only serve as the target of message sending and receiving, but also as relay nodes to forward messages [27]. Researchers in the field of V2X communications have proposed various probability-based multi-hop broadcasting schemes. As in the weighted p-persistent broadcast (WPB) scheme proposed in reference [11], each vehicle that receives the message within the reference transmission range *R* of the sending node rebroadcasts it with probability *P_Forward_*. The probability is proportional to the Euclidean distance *d* from the current vehicle to the sending vehicle and can be expressed as:

(1) $$P_{\mathrm{Forward}}=\frac{d}{R}$$

This is a fundamental probability-based forwarding scheme that assigns higher forwarding probabilities to more distant nodes, thereby enabling the message to be transmitted farther with each hop.

The CBR-based Probabilistic Forwarding Protocol (CPF) proposed in [23] utilizes the distance between the receiver and sender along with the CBR to determine the forwarding probability of candidate forwarding nodes, as expressed by following equation.

(2) $$P_{\mathrm{Forward}}=\{\begin{array}{c} exp\left(-\frac{R-d\left(x\right)}{10\sigma }\cdot \mathrm{CBR}\left(x\right)\right),d(x)\leq R\\ 1,\;d(x)>R \end{array},$$

Equations (1) and (2) indicate that the transmission radius *R* is required when calculating the forwarding probability *P_Forward_*. In practice, *R* is typically set as a fixed reference value (such as 300 m, 500 m). However, during the actual transmission process of wireless communication, the effective single-hop transmission radius of each vehicle is determined by multiple factors such as transmit power, physical layer channel conditions, and the modulation and demodulation schemes [28]. Consequently, it may exceed the reference value *R*. At this point, the forwarding probability of the candidate forwarding nodes that successfully receive the message at the *R* boundary and outside the boundary will both be 1. Therefore, forwarding redundancy will occur at the boundary *R* and beyond, increasing the probability of transmission collisions.

**Figure 2:**
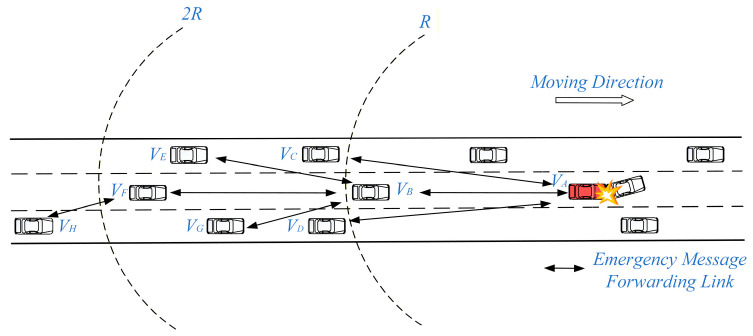
Multi-hop communication for V2V for highway scenarios.

Next, we will briefly analyze the issue of redundant forwarding of Emergency messages at boundary positions in schemes such as WPB and CPF, using the multi-hop communication scenario for C-V2X in Figure 2. Assume that the reference transmission radius *R* of wireless communication for each vehicle is 500 m. At time *T*, *d_BA_* = 480 m (distance between *V_B_* and *V_A_*), *d_CA_* = 510 m, *d_DA_* = 510 m, *d_EA_* = 900 m, *d_FA_* = 970 m, and *d_GA_* = 750 m. For all rear-following except the accident vehicle *V_A_*, the probability of forwarding the Emergency message is calculated using the probabilistic model in Equation (2). During the process of message forwarding, channel resource selection of each vehicle is performed based on C-V2X Mode 4.

**Figure 3:**
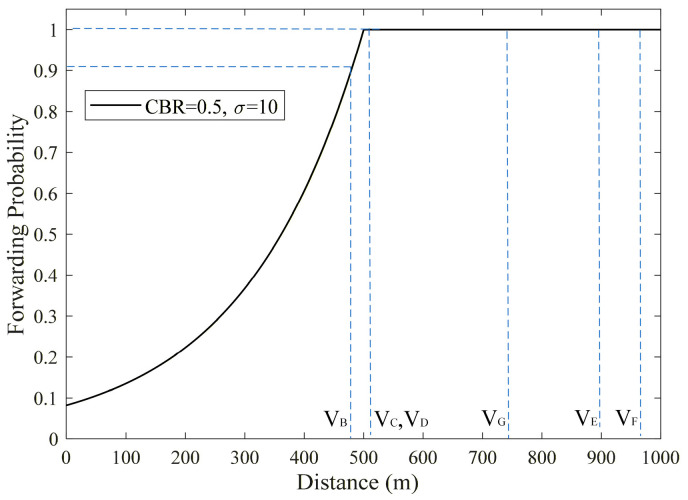
Forwarding Probability Distribution Curve in CPF Scheme.

When a vehicle *V_A_* is involved in a rear-end collision, an Emergency message must be immediately transmitted by *V_A_* to other vehicles behind to alert them to change lanes in advance for emergency evasion. Among vehicles within the transmission boundary *R*, vehicle *V_B_* is the farthest from *V_A_*. Therefore, *V_B_* has the highest probability of serving as the relay node to forward this message. The actual single-hop transmission radius of a vehicle is determined by multiple factors, such as transmit power, physical layer channel conditions, and modulation and demodulation schemes. Therefore, its actual transmission distance may exceed the reference value of *R*. When vehicles *V_C_* and *V_D_*, which are close to the boundary range of *R* but out of *R*, successfully receive the Emergency message sent by *V_A_*, since their distances *d_CA_* and *d_DA_* from *V_A_* are both greater than *R*, *V_C_* and *V_D_* will forward the Emergency message with probability of 1, as shown in Figure 3. At this point, *V_B_*, *V_C_*, and *V_D_* may all act as forwarding nodes to relay the Emergency message, potentially causing forwarding redundancy of the Emergency message. In the process of resource selection based on C-V2X Mode 4, *V_B_*, *V_C_*, and *V_D_* will have resource selection windows with large overlapping ranges, because they receive messages and perform resource selection almost simultaneously, thereby increasing the probability of resource selection conflicts, increasing the collision probability of Emergency messages, and reducing the reliability of multi-hop transmission.

Especially in road scenarios with high vehicle density, excessive forwarding redundancy increases the transmission burden on C-V2X wireless channels, leading to a high probability of resource selection conflicts. The interference caused by forwarding redundancy from numerous vehicles may cause receiving vehicles to fail to correctly decode received packets due to insufficient Signal-to-Interference-plus-Noise Ratio (SINR) [29]. Furthermore, in highway scenarios where the channel condition is relatively good, the actual single-hop transmission range may exceed 2*R*, which may result in even more additional forwarding redundancy in probability-based forwarding schemes. In probability-based forwarding schemes, nodes near the boundary *R* position are usually assigned higher forwarding expectations due to their greater distance from the sending node. Therefore, reducing forwarding conflicts at boundary positions is particularly important.

On the other hand, if nodes outside the boundary *R* can perform forwarding while reducing forwarding redundancy and lowering the probability of forwarding collisions, then nodes beyond the boundary can be effectively utilized to execute longer-distance single-hop forwarding. This facilitates increasing single-hop transmission distances and reducing forwarding hop count. For example, when the sending node *V_A_* forwards the Emergency message without relying on external nodes beyond boundary *R*, the transmission path from *V_A_* to *V_H_* requires at least three hops (*V_A_* ⟶ *V_B_* ⟶ *V_F_* ⟶ *V_H_*). If forwarding is performed by a node out of boundary *R*, assuming the *V_G_* receives the Emergency message from *V_A_*, then *V_G_* can act as a forwarding node, and the transmission path from *V_A_* to *V_G_* requires only two hops (*V_A_* ⟶ *V_G_* ⟶ *V_H_*).

In summary, to address the issue of redundant forwarding at the boundary of the transmission range of the sending vehicle, it is necessary to further optimize the forwarding mechanism beyond the boundary in Probabilistic forwarding schemes like WPB and CPF. This optimization aims to reduce the forwarding collision probability of the Emergency message at boundary *R* and outside boundary *R*, while effectively utilizing nodes outside the boundary for forwarding. Subsequently, some relevant mathematical models will be established to conduct a quantitative analysis of the aforementioned redundant forwarding problem at the boundary.

### 3.3. Multi-Hop Broadcast Forwarding Collision Probability Model Based on C-V2X Mode 4

#### 3.3.1. Mathematic Model of Forwarding Collision Probability Related to the Number of Forwarding Nodes

In C-V2X communication systems, Emergency messages are typically triggered by specific events and require multi-hop transmission. The multi-hop broadcast of Emergency messages may trigger resource reselection in C-V2X Mode 4 because the channel resources pre-allocated to Beacon messages cannot meet the transmission requirements of Emergency messages [30]. During the resource reselection process, the multi-hop broadcast of Emergency messages may cause the selection windows to overlap, increasing the collision probability of the Emergency messages themselves and subsequent Beacon messages.

This section primarily addresses the issue of the increased forwarding collision probability caused by redundant forwarding of Emergency messages outside the boundary *R*. Therefore, certain assumptions are required to simplify the establishment of the collision probability model. Let the total number of subframes in the resource selection window be *NSF*, and the number of subchannels in each subframe be *N_SC_*. Then the total number of available subchannels in the resource selection window is $M_{\mathrm{total}}=N\mathrm{SF}\times \mathrm{Nsc}$, the number of subchannels in the final candidate resource set *S_B_* is ${NC=0.2\times M}_{\mathrm{total}}$. According to reference [31], for a given relaying vehicle node *V_k_*, the collision probability of message transmission with any other surrounding vehicle node *V_i_* can be expressed as:

(3) $$P_{\mathrm{col}\;}\left(V_{k,i}\right)=P_{s}\left(V_{k,i}\right)\cdot \frac{C_{C}\left(V_{k,i}\right)}{N_{C}^{2}}$$

where *Nc* is the number of candidate resources, *C_C_*(*V_K,i_*) denotes the number of common CSRs in the final candidate resource set *S_B_* for nodes *V_k_* and *V_i_*. *C_C_*(*V_K,i_*) is a function of the distance between the two nodes; this paper assumes it to be a known value. *P_S_*(*V_k,i_*) represents the probability that vehicle *V_k_* cannot perceive the resources reserved by other vehicles when performing resource selection.

In the *h*-th hop forwarding, the probability of transmission collision between forwarding node *V_k_* and at least one other forwarding node is:

(4) $$P_{C}^{h}=1-\prod_{V_{i}\in V_{\mathrm{CF}}^{h}}^{Z^{h}-1}\left(1-P_{\mathrm{col}}\left(V_{k,i}\right)\right)$$

where *Z^h^* denotes the number of forwarding nodes in the *h*-th hop transmission, which is the size of the forwarding node set of $V_{\mathrm{CF}}^{h}.$ From the above probabilistic model, it can be observed that as the number of forwarding nodes increases, the collision probability in Emergency message forwarding between these nodes also rises. Therefore, during the forwarding process of Emergency messages, it is necessary to optimize the forwarding probability beyond the boundary and control the number of forwarding nodes outside the boundary.

The above analysis shows that controlling the number of forwarding nodes at each hop is crucial for reducing forwarding redundancy and improving the reliability of multi-hop transmission of Emergency messages. In the multi-hop broadcast schemes based on probabilistic forwarding, the forwarding probability of a node influences its forwarding behavior. During the *h*-th hop forwarding, if nodes both within and outside the coverage boundary of *h*-th hop forwarding all have high forwarding probabilities, this may lead to an excessive number of forwarding nodes near the boundary position, potentially causing channel resource selection conflicts among these nodes and increasing collision probability of the Emergency messages. Next, we will establish a mathematical model that relates the impact of forwarding probability on the number of forwarding nodes and conduct a quantitative analysis.

Assume that a group of vehicles is traveling at constant speed along a straight road with a length of *L*, and the vehicles are uniformly distributed on the road. The vehicle density is ρ, and its unit is the number of vehicles per meter. Each vehicle can receive messages normally and calculates a forwarding probability based on distance *x* to determine whether to forward the message. The reference transmission radius for vehicle nodes is *R*. An Emergency message is broadcast by the sending vehicle toward the opposite direction of their travel.

The number of forwarding nodes in the *h*-th hop during the multi-hop broadcast of an emergency message can be approximately estimated by using the following formula.

(5) $$Z_{h}=\rho \cdot L_{h}\cdot {\overset{-}{P}}_{h}$$

where *L_h_* is the length of the road covered by the *h*-th hop. For a complete hop, $L_{h}=R$, and for the last hop near the end of the road, $L_{h}=L-\left(h-1\right)R$. ${\overset{-}{P}}_{h}$ is the average forwarding probability of nodes within the coverage of the *h*-th hop. From the above equation, it can be seen that when ρ and *L_h_* are constant, *Z_h_* is linearly proportional to ${\overset{-}{P}}_{h}$. Therefore, the number of forwarding nodes in the *h*-th hop can be adjusted by modifying the average forwarding probability of the *h*-th hop. Taking the CPF scheme as an example, ${\overset{-}{P}}_{h}$ in Equation (5) can be calculated as:

(6) $${\overset{-}{P}}_{h}=\{\begin{array}{c} \frac{1}{R}\int_{0}^{R}\exp\left(-\frac{R-x}{10\sigma }\cdot \mathrm{CBR}\right)\mathrm{dx},\;h=1,\;x\leq R\\ 1,\;h>1,x>R \end{array},$$

Substituting the expression for $Z_{h}$ into Equation (4) yields:

(7) $$P_{C\;}^{h}=1-\prod_{V_{i}\in V_{\mathrm{CF}}^{h}}^{\rho \cdot L_{h}\cdot {\overset{-}{P}}_{h}-1}\left(1-P_{\mathrm{col}}\left(V_{k,i}\right)\right)$$

As can be readily seen from Equations (6) and (7) that reducing ${\overset{-}{P}}_{h}$ can effectively decrease $Z_{h}$ (the number of forwarding nodes at the *h*-th hop), thereby lowering $P_{C}^{h}$ (the message collision probability among forwarding nodes at the *h*-th hop).

#### 3.3.2. Mathematic Model of Forwarding Collision Probability Related to Distance

During the process of multi-hop broadcast of Emergency messages, any single-hop transmission collision is considered a transmission collision occurring during multi-hop transmission. When a vehicle node *V_t_* transmits data packets using the same C-V2X wireless channel as a neighbor vehicle node *V_i_*, the resulting interference may cause the receiving vehicle *V_r_* to fail to correctly decode data packets due to insufficient SINR. This kind of transmission error is termed message forwarding collision error, which depends on factors such as communication link performance, the sensing-based SPS scheme defined in C-V2X Mode 4, and the distances between vehicles *V_t_*, *V_r_*, and *V_i_*. As indicated in reference [31], the collision probability $P_{\mathrm{COL}}\left(d_{t,r}\right)$ can be calculated as follows:

(8) $$P_{\mathrm{COL}}^{i}\left(d_{t,r}\right)=\prod_{i}(1-P_{\mathrm{COL}}^{i}\left(d_{t,r},d_{t,i},d_{i,r})\right)$$

The collision probability $P_{\mathrm{COL}}^{i}\left(d_{t,r},d_{t,i},d_{i,r}\right)$ of node *V_t_* sending a message to node *V_r_* due to interference signals from *V_i_* can be expressed as:

(9) $$P_{\mathrm{COL}}^{i}\left(d_{t,r},d_{t,i},d_{i,r}\right)\;={\;P}_{\mathrm{SIM}}\left(d_{t,i}\right)\cdot P_{\mathrm{INT}}\left(d_{t,r},d_{i,r}\right)$$

where $P_{\mathrm{SIM}}\left(d_{t,i}\right)$ is the probability that *V_t_* and *V_i_* transmit using the same channel resources. $P_{\mathrm{INT}}\left(d_{t,r},d_{i,r}\right)$ represents the probability that the interference generated by *V_i_* on receiver node *V_r_* exceeds a certain threshold. Interference exceeding this threshold may cause receiver node *V_r_* to fail to correctly decode data packets due to insufficient SINR. Apparently, interfering node *V_i_* will generate a higher power interference signal when *V_i_* is closer to the sending node *V_t_* or the receiving node *V_r_*, thus increasing the $P_{\mathrm{INT}}\left(d_{t,r},d_{i,r}\right)$ and causing a higher probability of transmission failure.

In probability-based forwarding schemes such as WPB and CPF, nodes located at or outside the boundary have forwarding probabilities approaching or equal to 1. These nodes are typically close to each other, so the interference signals they generate will have a greater impact on each other and will more easily lead to increased collision probability of message transmission at the boundary position of the transmission range of the sending node.

## 4. The Proposed Schemes

### 4.1. Description of Proposed Schemes

The theoretical analysis in the previous section shows that it is necessary to alleviate the forwarding redundancy in the probabilistic forwarding schemes when the actual transmission distance of the vehicle sending the Emergency message is greater than the reference transmission range *R*, to reduce message transmission collisions at the location of boundary *R*. This paper further proposes two boundary-optimized probabilistic forwarding schemes based on the CPF scheme.

(1) CPF Scheme with Boundary Probability Optimization (CPF-BPO): It utilizes parameters such as the distance *d*(*x*) between the receiver and sender and the CBR to determine the forwarding probability of candidate forwarding nodes. Vehicle nodes outside the boundary replace *d*(*x*) with the value obtained by taking the modulus operation of *d*(x) modulo *R*, ensuring identical forwarding probability distributions both inside and outside the radius *R* of the sending node. This reduces the number of forwarding nodes and redundant forwarding at the boundary *R* and beyond, while lowering the probability of resource selection conflicts for nodes at the boundary *R* and beyond.
(2) CPF Scheme with Boundary Probability Optimization and Delay (CPF-BPOD): It performs the same forwarding probability calculation process as CPF-BPO, and additionally introduces a backoff timer for nodes outside boundary *R* of the sending node. When the period of the backoff timer expires, if the node has not received the next hop forwarding for the same Emergency message, it executes the current forwarding; otherwise, it cancels the forwarding. The backoff timer serves to further reduce forwarding redundancy of messages outside boundary *R* compared with CPF-BPO. The backoff timer duration can be set appropriately based on the single-hop forwarding delay of the emergency message.

The forwarding probability calculation model in the CPF-BPO and CPF-BPOD schemes is expressed as follows:

(10) $$P_{\mathrm{Forward}}=\exp\left(-\frac{R-\mathrm{mod}\left(d(x),R\right)}{10\sigma }\cdot \mathrm{CBR}\left(x\right)\right)$$

where *mod*(*d*(*x*), *R*) denotes *d*(*x*) modulo *R*, *d*(*x*) represents the distance between the current node and the sending node, *R* is the reference transmission radius of each vehicle node, *CBR*(*x*) is the latest CBR value measured by vehicle node *x*, and σ ≥ 1 is an integer coefficient used to adjust the shape of the forwarding probability curve. The forwarding probability distribution outside the boundary *R* under the CPF-BPO scheme remains consistent with that inside boundary *R*. This facilitates controlling the number of forwarding nodes outside boundary *R* based on the CPF scheme, thereby reducing forwarding redundancy of Emergency messages outside boundary locations and lowering the transmission collision probability of Emergency messages.

The calculation formula for the backoff timer assigned to nodes outside boundary *R* in the CPF-BPOD scheme is as follows:

(11) $$T_{\mathrm{backoff}}=\left\lfloor \frac{d\left(x\right)}{R}\right\rfloor \cdot \tau$$

where ⌊·⌋ is the floor sign. And τ is the unit backoff time, which can be set to an appropriate value (such as 10 ms, 15 ms) based on the single-hop transmission delay of the message. As can be seen from the above formula, the backoff timer length increases by τ every time the distance between the current node and the sending node exceeds double *R*. The purpose of this backoff timer is to further reduce forwarding redundancy outside the boundary *R*.

### 4.2. The Effectiveness of Proposed Schemes

**Figure 4:**
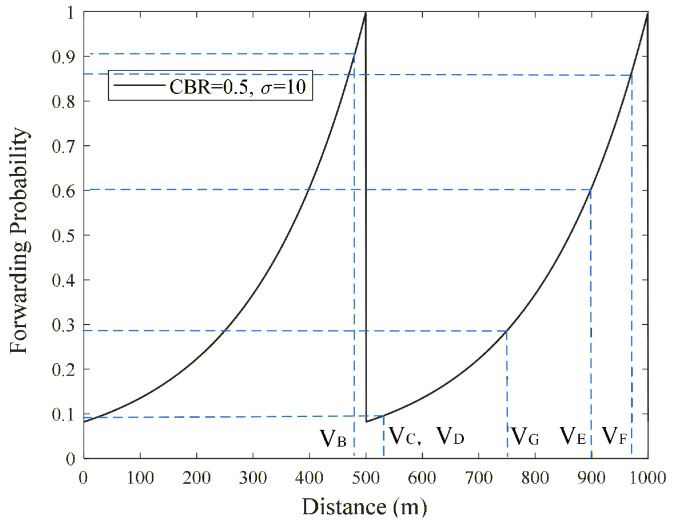
Forwarding Probability Distribution Curve in CPF-BPO and CPF-BPOD Schemes.

The effectiveness of the proposed schemes in addressing the issue of boundary forwarding redundancy will be analyzed in conjunction with Figure 2 above. When candidate nodes both within and outside the boundary *R* successfully receive Emergency messages from *V_A_*, their forwarding probability is calculated by Equation (10). As shown in Figure 4, the forwarding probability of vehicle nodes located within and close to boundary *R* is much higher than that of vehicle nodes located outside and close to boundary *R*. This helps reduce redundant forwarding outside the boundary *R* and lowers the probability of nodes near the boundary selecting the same channel resource when performing resource selection.

As shown in Figure 2 above, vehicle *V_A_* was involved in a rear-end collision at time *T*. Therefore, an Emergency message must be immediately broadcast to the following vehicles to alert them to change lanes in advance for emergency evasion. When vehicle *V_B_* receives the Emergency message broadcast by vehicle *V_A_*, since *V_B_* is the farthest from *V_A_* within the range of *R*, *V_B_* has the highest probability of forwarding the Emergency message. Assume that the actual single-hop transmission distance of the message exceeds the reference value *R*, when vehicle nodes *V_C_* and *V_D_* (located near the boundary of *R* but outside the range of *R*) successfully receive the Emergency message sent by *V_A_*, both *V_C_* and *V_D_* calculate their forwarding probabilities by using their distances *d_CA_* and *d_DA_* from *V_A_* to perform the modulo operation on *R*.

Since nodes *V_B_*_,_ *V_C_*, and *V_D_* are close to each other, they receive the Emergency message sent by *V_A_* almost simultaneously. However, as shown in Figure 4, *V_B_* has a much higher forwarding probability than *V_C_* and *V_D_*. Specifically, the forwarding probability *P_B_* for *V_B_* is approximately 90%, while the forwarding probabilities *P_C_* and *P_D_* for *V_C_* and *V_D_* are approximately 8%. Therefore, at time *T*, *V_B_* has a high probability of acting as the forwarding node within boundary *R*, while *V_C_* and *V_D_* have a very low probability of forwarding the Emergency message, which is beneficial to reducing the forwarding redundancy of Emergency messages and the probability of channel resource selection conflicts of vehicle nodes near the boundary *R*.

On the other hand, CPF-BPO and CPF-BPOD, by optimizing the forwarding probability outside the boundary *R*, can reduce forwarding redundancy of Emergency messages near the boundary while assigning higher forwarding probabilities to vehicle nodes further outside the boundary. This is conducive to increasing the single-hop transmission distance of Emergency messages and reducing the multi-hop transmission delay of Emergency messages. As shown in Figure 2 above, assuming that *V_F_* also receives the Emergency message from *V_A_*, it calculates the forwarding probability based on the value of *d_FA_* modulo *R*. In this case, *V_F_* still has a high forwarding probability (Figure 4 shows that the forwarding probability of vehicle *V_F_* is approximately 86%). Therefore, *V_F_* may directly forward the Emergency message as a forwarding node outside the boundary *R*, which can help increase the effective single-hop transmission distance of the Emergency message and reduce the number of forwarding hops. For example, when utilizing vehicles outside boundary *R* (such as vehicle *V_F_*) for forwarding, the path *V_A_* transmits the Emergency message to *V_H_* probably only via two hops (*V_A_* ⟶*V_F_* ⟶*V_H_*). Furthermore, since *V_F_* is located farther from the boundary *R*, even if it causes interference to vehicles near boundary *R*, the interference power remains relatively low, thus reducing the probability of message transmission failure.

Next, the role of the backoff timer in the CPF-BPOD scheme will be analyzed. Suppose that node *V_E_* outside the boundary starts a backoff timer after receiving the Emergency message from node *V_A_*. Within the time range [*t*, *t* + *T_backoff_*_,*E*_] of the backoff timer, *V_E_* receives the same Emergency message again from node *V_B_*. *V_E_* will cancel the forwarding it is waiting for and update its own probability and backoff timer based on the message received from *V_B_*. If *V_E_* does not receive a message from another node after the backoff timer expires, it will perform a forwarding operation. The backoff timer *T_backoff_*_,*E*_ of *V_E_* is calculated according to Equation (11). Therefore, the backoff timer mechanism in the CPF-BPOD scheme could reduce forwarding redundancy of Emergency messages at boundary positions and lower the forwarding collision probability.

In summary, the two probability-based forwarding schemes proposed in this paper can effectively reduce the forwarding redundancy of Emergency messages at and outside the boundary *R* by optimizing the forwarding probability distribution outside the boundary *R* and allocating backoff timers to nodes outside the boundary. This can alleviate the increase in the collision probability of Emergency messages and enhance the reliability of C-V2X multi-hop communication.

Algorithm 1 presents the detailed pseudocode implementation of the probabilistic forwarding scheme with boundary optimization. The sending node assigns a unique ID to each message when transmitting an Emergency message. Forwarding nodes do not alter the ID of Emergency messages during transmission. Nodes determine whether they have received an Emergency message for the first time based on the *Msg*(*ID*) of the received message. The forwarding probability of candidate nodes is calculated by Equation (10). Based on the current forwarding hop-count (*CurTransHop*) cached by the forwarding node and the forwarding hop-count (*MsgHop*) specified in the Emergency message, the node decides whether to cancel the current waiting forwarding or update the backoff timer.
**Algorithm 1:** Probabilistic Forwarding Scheme with Boundary Optimization based on C-V2X Mode 4Notations:*Msg*(*ID*): each Emergency message has a unique ID;*CurTransHop*: The number of forwarding hops cached by the current node from the first receipt of the Emergency message, or the default value is 1;*MsgHop*: The number of forwarding hops in the currently received *Msg*(*ID*);Input: Emergency message;Output: forwarding probability and whether to execute forwarding;*V_X_* receives a *Msg*(*ID*) from *V_S_*; **if** *V_X_* receives *Msg*(*ID*) for the first time  **if** *V_X_* is within the road range of interest    Calculate the forwarding probability *P_forward_* by Equation (10);    Calculate the backoff timer by Equation (11);    **if** uniform(0,1) < *P_forward_*
     Execute resource selection for *Msg*(*ID*) and wait for forwarding;**else**  **if** *CurTransHop* + 1 <= *MsgHop*     **if** *V_X_* receives the same *Msg*(*ID*) within backoff timer     Cancel forwarding and delete the Grant of *Msg*(*ID*);  **else** *CurTransHop* <= *MsgHop*    Update the forwarding probability by Equation (10);    Update the backoff timer by Equation (11);    Update the *CurTransHop*;End

## 5. Simulation Results

In this section, the proposed CPF-BPO and CPF-BPOD are simulated and compared with other compared schemes. Several different metrics are used to evaluate the single-hop performance of Beacon messages and the multi-hop performance of Emergency messages, respectively.

### 5.1. Simulation Configurations

The simulation experiments are based on Veins, which is an open-source framework for vehicle network simulation [32]. Different road traffic environments are established on SUMO [33]. The OMNET++, which is a network simulation tool, is used to simulate the communication performance of different schemes under specified road traffic conditions [34]. The simulation assumptions and parameters are set according to [35]. This paper focuses on the boundary redundancy forwarding problem of probabilistic forwarding schemes under different vehicle densities or message traffic, especially when the vehicle density is high. A simple straight 4 km road with six lanes was set up as the traffic simulation scenario in the simulation experiment. Because compared with the complex urban road scenarios, straight road scenarios make it easier to control the traffic vehicle density during simulation and facilitate the analysis of message transmission performance under different vehicle densities in conjunction with theory. Although theoretically, the probabilistic forwarding scheme proposed in this paper does not rely on the road topology to calculate probabilities, and the different traffic vehicle densities on straight roads can be compared to vehicle distribution in urban scenarios, the applicability of the proposed scheme to more complex scenarios such as urban environments may still require further exploration.

The Beacon message is set to 190 bytes in length and occupies 1 sub-channel, while the Emergency message is set to 512 bytes in length and occupies 3 sub-channels. Each vehicle periodically broadcasts Beacon messages at 100 ms intervals, and the maximum tolerable delay for Beacon messages is 100 ms. Every second, vehicles positioned at the head or rear of the road initiate an Emergency message to transmit in the opposite direction of driving. The maximum transmission distance of Emergency messages is limited to 3.8 km, and the maximum tolerable delay for each hop is 20 ms. The single-hop reference transmission radius of each vehicle is 500 m. Table 1 provides detailed parameter configurations.

The following seven metrics are used to evaluate the single-hop communication performance of vehicle Beacon messages and the multi-hop communication performance of Emergency messages.

**(a)**  S**ingle-hop Performance Metrics:**
  - Packet Delivery Ratio (PDR): The ratio of the number of successfully received packets to the total number of packets sent.
  - Packet Collision Ratio (PCR): The ratio of the number of packets lost due to transmission collision to the total number of packets sent.
  - Packet Inter-Reception (PIR): The time interval between two consecutive packets received from the same vehicle.
**(b)**  **Multi-hop Performance Metrics:**
  - Multi-hop Packet Delivery Ratio (MPDR): The ratio of emergency messages forwarded to 3.8 km away to the total number of Emergency messages sent.
  - Forwarding Hop-count: The cumulative number of hops for message forwarding from the sending node to the final node at the end of the road.
  - Reliability Factor: The ratio of the average number of unique Emergency messages received by nodes within the range of multi-hop transmission to the total number of Emergency messages sent.
  - Forwarding Ratio: The average ratio of the number of times a node forwards an Emergency message to the number of unique Emergency messages it receives.
**(c)**  **Schemes Comparison and Analysis**
  - Single-hop: There are only single-hop broadcast Beacon messages in the network, and no emergency messages. The transmission of Beacon messages is not affected by emergency messages. This scheme serves as the baseline for comparative analysis when evaluating the impact of the multi-hop broadcast of the Emergency message on the transmission performance of the Beacon message.
  - Flooding: Emergency messages are multiple-hop broadcast by flooding, wherein all nodes function as forwarding nodes to execute forwarding of Emergency messages. This approach represents the simplest multi-hop broadcast protocol and serves as the baseline scheme for multi-hop performance analysis.
  - CPF (CBR-based Probabilistic Forwarding Protocol): Emergency messages are multiple-hop broadcast by probability-based forwarding, and the forwarding probability is calculated by Equation (2). This scheme is used for comparative analysis with CPF-NFBB, CPF-BPO, and CPF-BPOD.
  - CPF-NFBB (CPF Scheme with Non-Forwarding Beyond the Boundary): Emergency messages are multi-hop broadcast with the CPF scheme, and nodes outside the transmission radius of the sending node do not participate in forwarding the Emergency messages. This scheme is used for comparative analysis with CPF-BPO and CPF-BPOD.
  - CPF-BPO (CPF Scheme with Boundary Probability Optimization): The proposed schemes of this paper, the forwarding probability of the candidate forwarding node is calculated by Equation (10). This scheme is used for comparative analysis with CPF, through which the optimization of performance for Emergency messages and Beacon messages in this scheme can be observed.
  - CPF-BPOD (CPF Scheme with Boundary Probability Optimization and Delay): The proposed scheme, based on the CPF-BPO scheme, allocates an additional backoff timer to the forwarding node outside of boundary *R*. Compared with CPF-BPO, we can observe the effectiveness of the backoff timer for reducing forwarding redundancy of Emergency messages.

### 5.2. The Impact of Multi-Hop Broadcasting of Emergency Messages on the Single-Hop Transmission Performance of Beacon Messages

**Figure 5:**
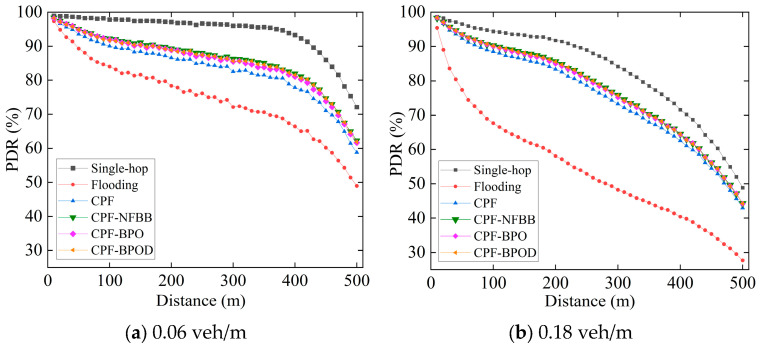
PDR of Beacon messages varying with distance under different vehicle densities.

Figure 5 illustrates the PDR of Beacon messages for several schemes under different vehicle densities. The PDR of Beacon messages in the Flooding scheme is significantly lower than that of the single-hop scheme at both vehicle densities, confirming that multi-hop broadcasting of Emergency messages adversely affects the transmission performance of Beacon messages. In the single-hop scheme, only single-hop Beacon messages are transmitted in the network, and there is no multi-hop transmission of Emergency messages. The single-hop performance of Beacon messages is not affected by the interference of Emergency messages. In the multi-hop scheme, the multi-hop transmission of Emergency messages will additionally increase the collision probability of Beacon messages, as described in the theoretical analysis in Section 3. Therefore, the single-hop scheme has the best PDR performance and is used as the benchmark to compare the impact of different multi-hop schemes on transmission performance of Beacon messages.

It can also be observed from Figure 5 that CPF-NFBB, CPF-BPO, and CPF-BPOD schemes have better PDR than CPF. This trend is also reflected in the average PDR values presented in Table 2, which demonstrates that controlling the number of forwarding nodes beyond the boundary *R* effectively reduces forwarding redundancy of Emergency messages, thereby reducing the transmission collisions between Beacon messages and Emergency messages and improving the transmission performance of Beacon messages.

CPF-NFBB achieves a slightly higher average PDR than CPF-BPO because it minimizes the forwarding number of Emergency messages by restricting their forwarding range within *R*. However, it completely blocks forwarding outside the boundary *R*, limiting the performance of multi-hop forwarding of Emergency messages. As described in Section 5.3, the MPDR and reliability factor of CPF-NFBB are slightly lower than those of CPF-BPO and CPF-BPOD, and the number of forwarding hops required is significantly higher than the latter two.

Although the PDR of the CPF-BPO scheme is slightly lower than that of CPF-NFBB, it represents an improvement over CPF. This is because CPF-BPO reduces forwarding redundancy of Emergency messages by optimizing the probabilistic forwarding mechanism outside boundary *R*, thereby lowering the collision probability of Beacon messages compared with the CPF scheme. In addition, compared with CPF-NFBB, CPF-BPO effectively utilizes nodes outside the boundary *R* to perform forwarding, thereby reducing the number of hops for Emergency message forwarding and lowering end-to-end delay. CPF-BPOD achieves a higher average PDR than CPF-BPO because it further reduces forwarding redundancy of Emergency messages by assigning backoff-timers to nodes outside boundary *R*. This facilitates a further reduction in the transmission collision probability between Emergency messages and Beacon messages compared with CPF-BPO, thereby enhancing the transmission success ratio of Beacon messages.

Table 2 lists the average PDR of Beacon messages in different schemes under different vehicle densities. Using CPF as a benchmark, it can be intuitively observed that CPF-BPO and CPF-BPOD both exhibit better PDR performance at vehicle densities such as 0.06 veh/m and 0.18 veh/m. Although the PDR of CPF-BPO and CPF-BPOD is slightly lower than that of CPF-NFBB, they still achieve a certain degree of performance improvement compared with CPF.

**Figure 6:**
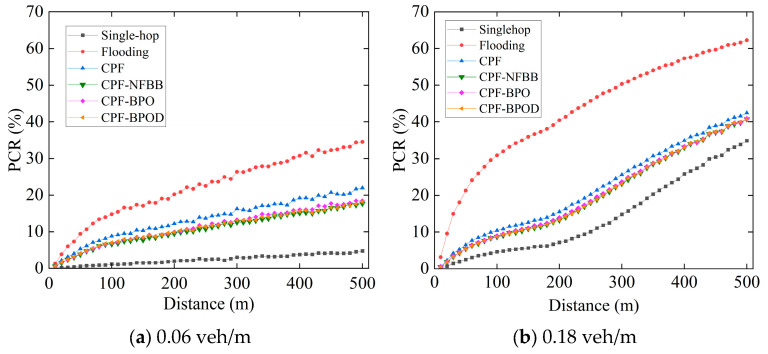
PCR of Beacon messages varying with distance under different vehicle densities.

Figure 6 shows the comparison of the PCR of Beacon message between several multi-hop broadcast schemes and the single-hop scheme. PCR evaluates the average packet lost rate caused by packet collisions, providing a more intuitive demonstration of transmission collisions of Beacon messages resulting from the multi-hop broadcast of Emergency messages. Firstly, the PCR of all schemes increases with the increase in vehicle density, because higher vehicle density leads to more Beacon messages transmitted in the network, making message transmission collisions more likely. Second, it is evident that the PCR of CPF under both vehicle densities is significantly higher than that of CPF-NFBB, CPF-BPO, and CPF-BPOD. This demonstrates that controlling the number of forwarding nodes outside the boundary effectively reduces transmission collisions between Emergency messages and Beacon messages, thereby lowering the packet collision probability of Beacon messages.

Table 3 lists the average PCR of Beacon messages in different schemes under different vehicle densities. CPF-NFBB has the lowest PCR in Table 3, as it restricts the message forwarding within *R*, thereby minimizing the number of forwarding messages by nodes out of the boundary to reduce the collision probability of messages. Although PCR of CPF-BPO is slightly higher than CPF-NFBB, it remains significantly lower than CPF. CPF-BPOD has a slightly lower PCR than CPF-BPO, because CPF-BPOD employs the backoff-timer mechanism to further reduce forwarding redundancy of Emergency messages.

**Figure 7:**
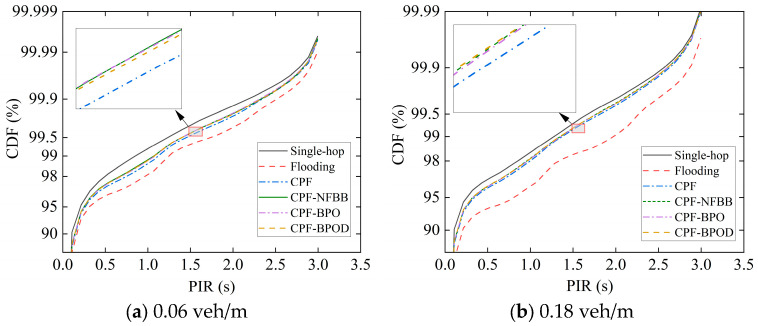
Cumulative distribution function of PIR of Beacon messages under different vehicle densities.

Figure 7 shows the Cumulative distribution function (CDF) of the Packet inter-reception interval (PIR) of Beacon messages in different schemes under different vehicle densities. To minimize the impact of vehicle mobility, only PIRs below 3 s are calculated. As can be seen from Figure 7, PIR ascends with the increase in vehicle density. Taking single-hop as an example, when vehicle densities are 0.06 veh/m and 0.18 veh/m, the CDF percentages with PIRs below 500 ms are 98.09% and 96.80%, respectively. This is because an increase in vehicle density leads to higher utilization of C-V2X channel resources, thereby increasing the probability of packet transmission collisions. When packets collide, they are lost, which increases the packet interval between consecutive data packet receptions.

Using CPF as the baseline, observing the CDF of PIR for schemes such as CPF-NFBB, CPF-BPO, and CPF-BPOD reveals that both the non-forwarding beyond boundary mechanism and the boundary probability optimization mechanism can improve PIR. CPF-NFBB improves PIR by completely prohibiting nodes outside the boundary from forwarding Emergency messages, thereby minimizing the packet loss due to collisions. The CPF-BPO and CPF-BPOD schemes can reduce the transmission collision caused by redundant forwarding for Emergency messages with a transmission distance exceeding *R*, thereby improving PIR.

### 5.3. Performance Analysis of Multi-Hop Broadcast of Emergency Messages

This subsection evaluates the multi-hop broadcasting performance of Emergency messages using metrics including the MPDR, reliability factor, forwarding hop count, and forwarding ratio. Detailed definitions of these evaluation metrics are provided in the simulation configurations of Section 5.1 above.

**Figure 8:**
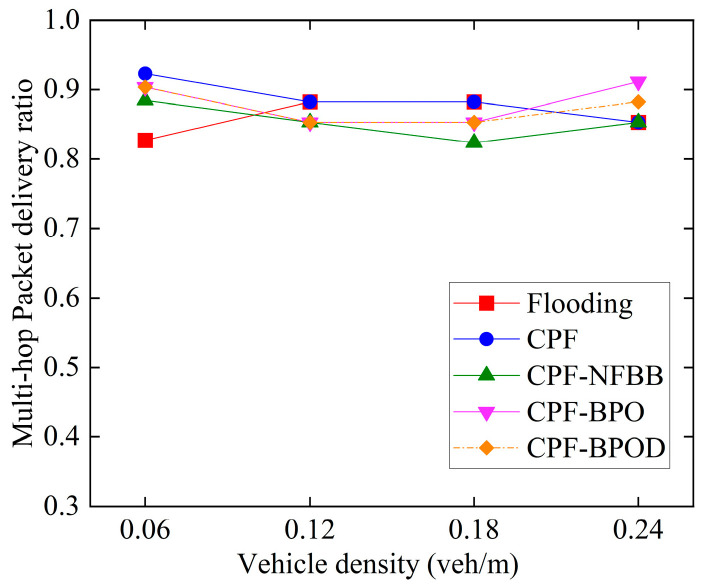
Multi-hop packet delivery ratio under various densities.

**Figure 9:**
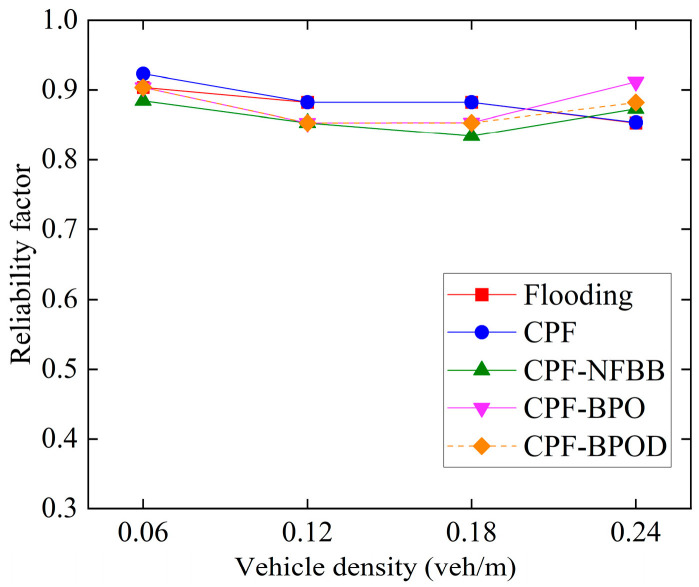
Reliability factors under various densities.

**Figure 10:**
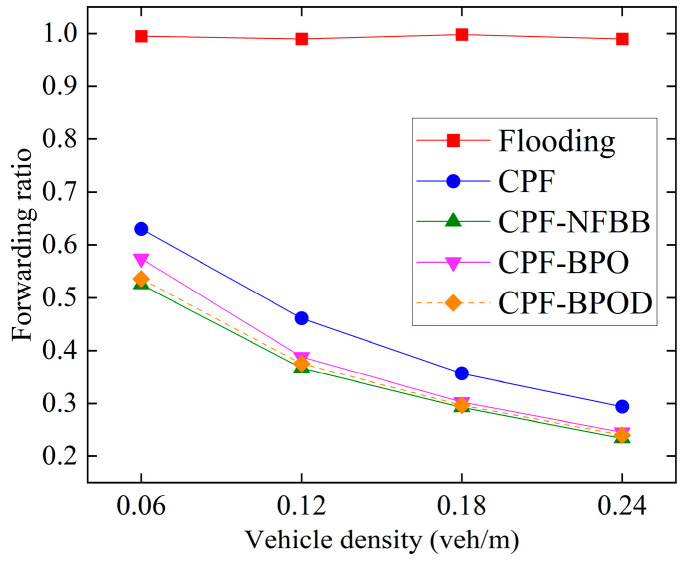
Forwarding ratio under various densities.

Figure 8 and Figure 9 illustrate the MPDR and reliability factors of several multi-hop broadcast schemes under different vehicle densities. First, comparing Flooding and CPF under different vehicle densities reveals that their MPDR and reliability factor values are similar. However, Figure 10 indicates that the forwarding ratio of the latter is significantly lower than that of the former. This demonstrates that the CPF scheme can effectively reduce forwarding redundancy by adjusting the number of forwarding nodes based on CBR, while maintaining a high MPDR and reliability factor, thereby lowering the transmission collision probability for Emergency messages. This is also the primary reason for the improved single-hop performance of CPF discussed in the previous subsection.

When vehicle density does not exceed 0.18 veh/m, the MPDR and reliability factors of CPF-NFBB, CPF-BPO, and CPF-BPOD are slightly lower than CPF. This is because probabilistic forwarding schemes with boundary optimization can reduce the forwarding ratio by optimizing the forwarding mechanism outside the boundary *R*, but this also limits the number of effective forwarding nodes, which is disadvantageous for improving the MPDR and the reliability factor at low vehicle densities. In contrast, CPF benefits from more abundant C-V2X wireless channel resources at low vehicle densities, resulting in a relatively low collision probability of Emergency messages. For the CPF scheme, nodes outside the boundary *R* that receive Emergency messages perform forwarding with probability 1, which helps to increase the number of forwarding by vehicles outside boundary *R* and improve the reliability of multi-hop forwarding of the Emergency messages. However, it will incur a loss in the performance of single-hop transmission. When vehicle density is high (such as 0.24 veh/m), the MPDR and reliability factors of the probabilistic forwarding schemes with boundary optimization are comparable to or better than CPF. Because a large number of forwarding nodes are available at higher vehicle densities, excessive redundant forwarding by nodes outside the boundary in the CPF scheme may lead to resource selection conflicts between nodes near the boundary, increasing the probability of collisions during Emergency message forwarding. When vehicle density is high, the decrease in reliability caused by this increased collision probability will outweigh the increase in reliability brought about by adding forwarding nodes. At this point, CPF-BPO and CPF-BPOD can control the number of redundant forwarding of Emergency messages to reduce the probability of collisions.

Figure 10 shows the forwarding ratio under different vehicle densities. Compared with Flooding, CPF significantly reduces the forwarding ratio by adjusting the number of forwarding nodes by assigning higher forwarding probabilities to more distant vehicle nodes, thereby minimizing forwarding redundancy of Emergency messages. The forwarding ratios of CPF-NFBB, CPF-BPO, and CPF-BPOD are noticeably lower than CPF, because these three schemes restrict the number of forwarding nodes outside the boundary *R*. A lower forwarding ratio implies fewer Emergency messages being forwarded and also indicates a smaller impact on the single-hop broadcast of Beacon messages, meaning better transmission performance of single-hop. CPF-BPO and CPF-BPOD have forwarding ratios close to CPF-NFBB while achieving multi-hop transmission performance comparable to or better than CPF.

**Figure 11:**
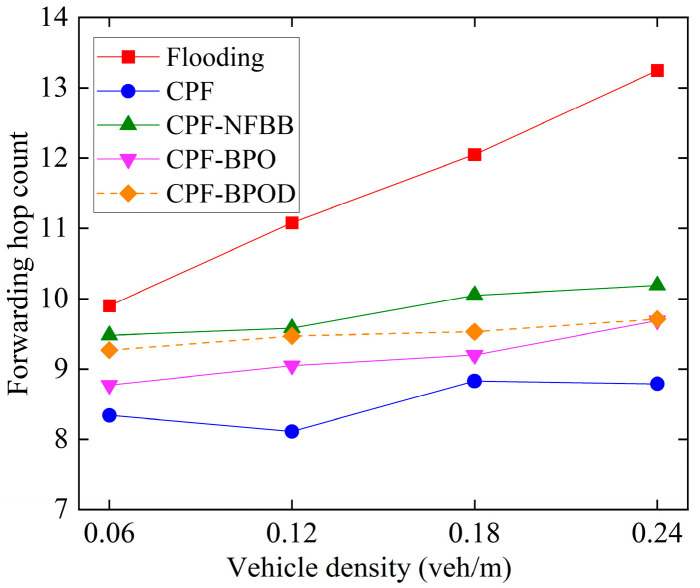
Forwarding hop count under various vehicle densities.

Figure 11 shows the forwarding hop count under different vehicle densities. First, compared with Flooding, CPF has a significantly lower average forwarding hop count. This is because in the Flooding scheme, all nodes that receive a message forward it with probability 1, which may accumulate a large number of forwarding hops. In contrast, the CPF scheme assigns a higher forwarding probability to nodes farther from the sending node, reducing the number of forwarding hops required for the message.

CPF-NFBB has a higher average forwarding hop count than CPF and CPF-BPO, because the CPF-NFBB scheme only allows nodes whose distance to the sending node is less than *R* to perform message forwarding. Therefore, CPF-NFBB may require more forwarding hops than CPF and CPF-BPO when the multi-hop transmission distances of these schemes are the same. Whereas CPF and CPF-BPO reserve the possibility of forwarding messages for nodes outside the boundary, which helps reduce the number of forwarding hops for Emergency messages transmitted across the boundary and reduces end-to-end delay. This is consistent with the theoretical analysis conclusions in Section 4.2.

CPF-BPOD has a slightly higher average forwarding hop count than CPF-BPO. This is because the backoff timer in the CPF-BPOD scheme may affect the forwarding path selection in the network, thereby suppressing the forwarding of nodes on some more optimal paths (with fewer forwarding hops). As a result, Emergency messages may be transmitted on paths that require more hops, thereby increasing the average forwarding hop count. However, as shown in Figure 11, the difference in the forwarding hop count between CPF-BPOD and CPF-BPO under different vehicle densities is only within one hop.

In addition, the distance-based backoff timer added in the CPF-BPOD scheme introduces additional transmission delay, but since the timer is only allocated to node outside the boundary, it will not increase delay significantly compared to having no timer.

In summary, the MPDR and reliability factors of the four algorithms in Figure 8 and Figure 9 are quite similar, indicating that the proposed schemes CPF-BPO and CPF-BPOD have performance related to multi-hop transmission reliability that is close to or slightly better than the comparative schemes. Meanwhile, as shown in Figure 10, the forwarding ratios of CPF-BPO and CPF-BPOD are significantly lower than that of CPF, which means that the former two effectively reduce forwarding redundancy and alleviate the performance degradation of single-hop transmission due to the interference caused by multi-hop forwarding of Emergency messages (as described in Section 5.2). As shown in Figure 11, compared with CPF-NFBB, CPF-BPO and CPF-BPOD have lower average forwarding hops, proving that effectively utilizing forwarding nodes outside the boundary can shorten the forwarding path. As shown in Table 4, combining the analyses of single-hop and multi-hop transmission performance in Section 5.2 and Section 5.3, it can be seen that the CPF-BPO and CPF-BPOD schemes can guarantee good MPDR and reliability factors, as well as good Beacon message transmission performance, while ensuring a lower forwarding ratio than CPF and fewer forwarding hop counts than CPF-NFBB.

CPF-BPO does not introduce additional backoff timer, it can better meet the transmission requirements of application services with high real-time requirements. Compared to CPF-BPO, CPF-BPOD can further limit redundant forwarding outside the boundary, but it also introduces an additional forwarding delay due to the backoff timer. Therefore, CPF-BPOD is more suitable for situations where the vehicle density is very high and the message traffic needs to be strictly controlled, as well as for application service transmission that is less sensitive to multi-hop end-to-end transmission delay.

### 5.4. Performance Analysis of CPF-BPO and CPF-BPOD with Different Transmission Radius

To further investigate the boundary optimization performance of the proposed probabilistic forwarding schemes, this section uses the CPF scheme as a baseline to examine the communication performance of Beacon messages and Emergency messages for CPF-BPO and CPF-BPOD with different reference transmission radius *R*.

**Figure 12:**
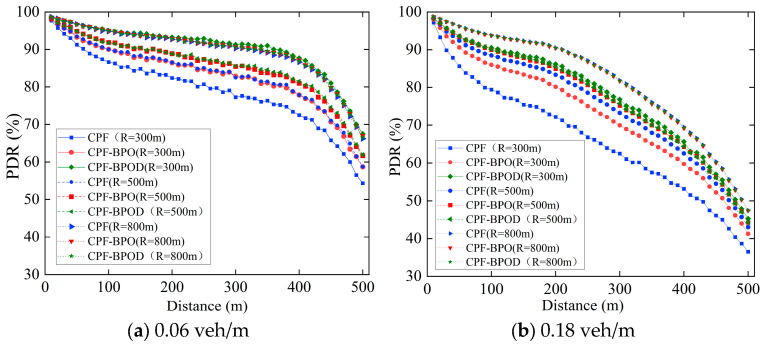
PDR of Beacon messages of three schemes with different *R* varies with distance under different vehicle densities.

Firstly, as shown in Figure 12, the PDR of Beacon messages decrease as transmission radius *R* shrinks under the same scheme. For instance, at a vehicle density of 0.18 veh/m, the PDR of CPF at *R* = 300 m is significantly lower than that at *R* = 800 m, which can also be found from the average PDR values across different vehicle densities and *R* values in Table 5. This indicates that a smaller reference transmission radius *R* results in more nodes outside the boundary *R*, leading to a higher number of nodes performing Emergency message forwarding. Consequently, the collision probability between Beacon messages and Emergency messages increases. This is consistent with the theoretical analysis presented in Section 3.3.

PDR curves in Figure 12 reveal that as the reference transmission radius *R* decreases, CPF-BPO demonstrates a more pronounced improvement in the PDR performance of the Beacon message, which can also be found in Table 5. For example, when the vehicle density is 0.06 veh/m and the *R* is 300 m, the average PDR of CPF is 79.10%, while that of CPF-BPO is 83.12%, representing an improvement of 4.02% over CPF. Furthermore, the average PDR of CPF-BPOD achieves an enhancement of 7.24% over CPF-BPO. This is because a smaller *R* may result in more forwarding nodes outside the transmission radius of the sending node, leading to a higher collision probability due to forwarding redundancy. Consequently, CPF-BPO and CPF-BPOD possess greater optimization potential.

When the *R* value is large, the PDR of CPF-BPO and CPF-BPOD approaches that of CPF. For instance, when the vehicle density is 0.06 veh/m and the *R* is 800 m, CPF achieves an average PDR of 89.57%, while the average PDR of CPF-BPO reaches 89.84%—an improvement of only 0.27% over CPF. The average PDR of CPF-BPOD achieves 89.77%, representing a mere 0.2% increase over CPF. A large transmission radius leads to fewer forwarding nodes outside boundary *R* compared with when *R* is smaller. At this point, all three schemes primarily rely on nodes within boundary *R* to forward Emergency messages. Consequently, the impact of multi-hop broadcast of Emergency messages on Beacon transmission performance is similar across the three schemes, resulting in similar PDR values in the three schemes.

**Figure 13:**
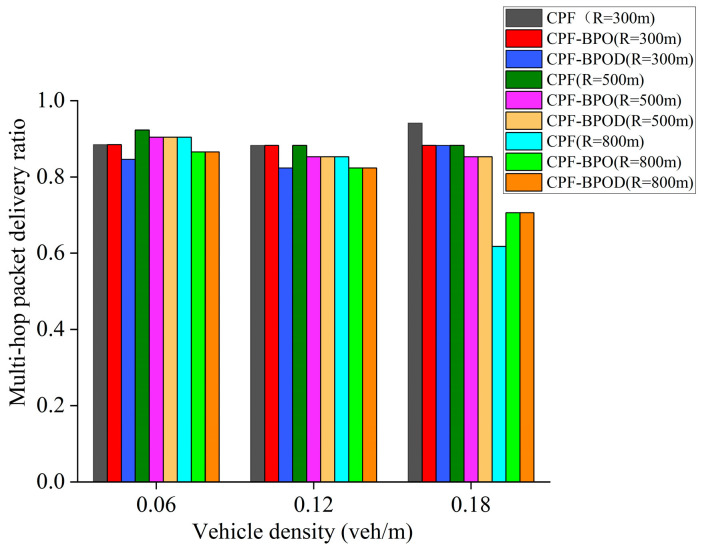
MPDR of three schemes with different *R* under various densities.

**Figure 14:**
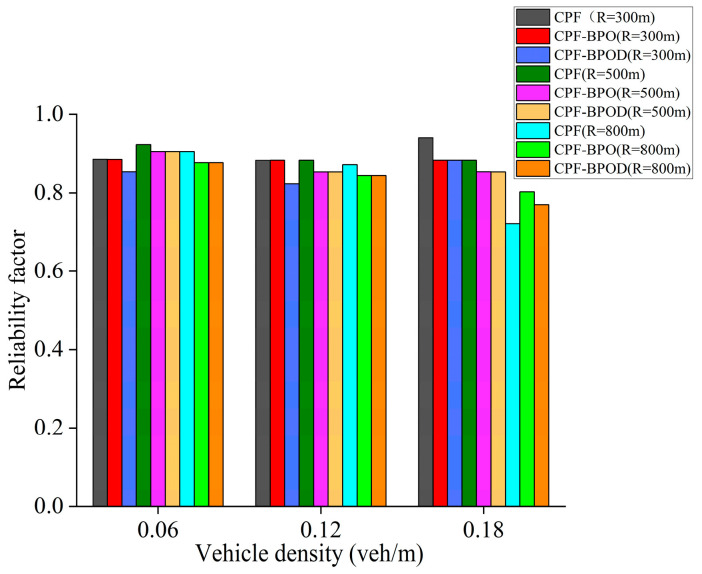
Reliability factors of three schemes with different *R* under various densities.

**Figure 15:**
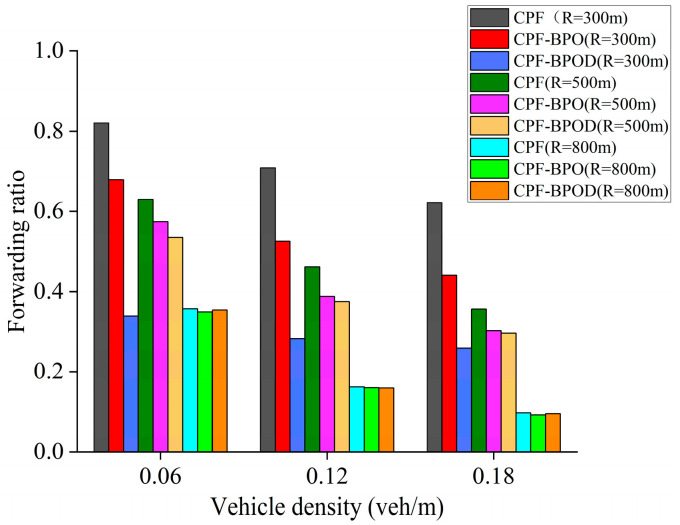
Forwarding ratios of three schemes with different *R* under various densities.

Figure 13, Figure 14 and Figure 15 show the MPDR, reliability factors, and forwarding ratio of the three schemes under different vehicle densities and *R*. CPF-BPO and CPF-BPOD have MPDR and reliability factors close to those of CPF. It can also be observed that the Forwarding ratios of CPF-BPO, CPF-BPOD, and CPF exhibit more pronounced differences when *R* is small (such as *R* = 300 m), and are significantly lower than CPF. This indicates that as the number of nodes outside the reference transmission radius *R* increases, CPF-BPO and CPF-BPOD demonstrate more pronounced effects in reducing the number of forwarding nodes and forwarding redundancy beyond the boundary through their boundary-optimized probabilistic forwarding mechanism.

When *R* is small, CPF-BPOD exhibits a reliability factor close to that of CPF-BPO while maintaining a significantly lower forwarding ratio. On the one hand, both schemes employ a probabilistic forwarding mechanism with boundary optimization to preserve the forwarding possibility for nodes receiving the Emergency message beyond boundary *R*, thereby maintaining the reliability of multi-hop transmission for Emergency messages. On the other hand, as *R* decreases, the number of forwarding nodes outside the boundary *R* increases. CPF-BPOD further reduces forwarding redundancy of Emergency messages beyond the boundary through its backoff timer, thereby achieving a more evident reduction in the Forwarding ratio.

When *R* is large (such as *R* = 800 m), the forwarding ratios of CPF-BPO and CPF-BPOD are close to that of CPF. Because the large communication radius provides wide-area coverage, the forwarding tendency of the CPF scheme outside the boundary is weakened due to the sparse number of forwarding nodes outside the boundary. In this scenario, the number of messages forwarded outside the boundary *R* of the three schemes is not much different, so the forwarding ratios are similar.

In summary, based on the analysis of results under different reference transmission radius, when the reference transmission radius *R* is small and the number of nodes located outside the boundary *R* is large, CPF-BPO and CPF-BPOD are more effective in reducing forwarding redundancy of Emergency messages and improving message transmission performance, and can maintain good single-hop and multi-hop transmission performance at the same time. When the *R* value is excessively large, although performance improvement is minimal, it does not introduce additional negative effects. This reduces the precision requirements for determining the value of *R* when calculating forwarding probabilities. Even if the reference transmission radius is set with poor rationality, its negative impact on forwarding probability calculations and message transmission performance can be minimized. In actual communication environments, the single-hop transmission radius of a vehicle is significantly influenced by the communication conditions (the single-hop transmission radius of a vehicle differs markedly between Line-of-Sight and Non-Line-of-Sight transmission). It is challenging to establish a universal and precise reference transmission radius *R* value. CPF-BPO and CPF-BPOD can effectively mitigate the negative impact of imprecise *R* values on forwarding probability calculations and message transmission performance in such scenarios.

## 6. Conclusions

This paper focuses on the boundary redundancy forwarding problem during multi-hop broadcasting of Emergency messages in C-V2X communication scenarios. Mathematical models relating forwarding collision probability to the number of forwarding nodes and to distance are established for quantitative analysis. To reduce forwarding redundancy and lower the forwarding collision probability of Emergency messages, two probability-based multi-hop broadcast schemes are designed: CPF-BPO and CPF-BPOD. Based on the Veins platform, the transmission performances of Beacon messages and Emergency messages in various schemes are compared and analyzed. Experimental results demonstrate that the proposed schemes, CPF-BPO and CPF-BPOD, can significantly reduce forwarding redundancy of Emergency messages compared with the CPF scheme and other schemes, while achieving superior multi-hop transmission performance, such as MPDR and reliability factor, along with better transmission performance of Beacon messages. Furthermore, when the reference transmission radius *R* is small, as the number of nodes outside of *R* increases, CPF-BPO and CPF-BPOD demonstrate more significant effects in reducing forwarding redundancy beyond the boundary through their boundary-optimized probabilistic forwarding mechanism. The main idea of the proposed schemes in this paper is to optimize the boundary problems of probabilistic forwarding schemes, which is theoretically applicable to most probabilistic forwarding schemes. However, this optimization still requires corresponding optimization design based on the specific algorithm ideas and processes of each different probabilistic forwarding scheme.

## Figures and Tables

**Table 1:** Simulation parameters.

**Parameters**	**Value**
Vehicle density	0.06, 0.12, 0.18, 0.24 veh/m
Road length	4 km
Number of lanes	3 lanes in each direction
Emergency message size	512 Byte
Beacon message size	190 Byte
Transmission interval	100 ms for Beacon, 1 s for Emergency
Maximum tolerable latency	100 ms for Beacon, 20 ms for Emergency
Carrier frequency	5.89 GHz
Channel bandwidth	10 MHz
Transmission power	23 mW
Reference transmission radius	500 m
Number of subchannels in each subframe	4
Size of subchannel	12 RBs
*P_keep_*	0
σ	10

**Table 2:** Average PDR of Beacon messages under different vehicle densities.

Vehicle Density	Single-Hop	Flooding	CPF	CPF-NFBB	CPF-BPO	CPF-BPOD
0.06 veh/m	94.25%	74.77%	83.37%	86.13%	85.49%	85.80%
0.18 veh/m	83.21%	54.24%	75.36%	77.21%	76.96%	77.17%

**Table 3:** Average PCR of Beacon messages varying with distance under different vehicle densities.

Vehicle Density	Single-Hop	Flooding	CPF	CPF-NFBB	CPF-BPO	CPF-BPOD
0.06 veh/m	2.42%	22.26%	13.15%	10.73%	11.31%	11.02%
0.18 veh/m	14.15%	43.41%	22.07%	20.20%	20.45%	20.24%

**Table 4:** Summary of part single-hop and multi-hop performance metrics.

Schemes with Different Vehicle Densities	Average PDR	MPDR	Reliability Factor	Forwarding Ratio	Forwarding Hop-Count
0.06 veh/m Flooding	74.77%	0.8239	0.9038	0.9952	9.90
0.06 veh/m CPF	83.37%	0.9230	0.9230	0.6300	8.34
0.06 veh/m CPF-NFBB	86.13%	0.8846	0.8845	0.5250	9.48
0.06 veh/m CPF-BPO	85.49%	0.9038	0.9038	0.5738	8.77
0.06 veh/m CPF-BPOD	85.80%	0.9038	0.9038	0.5353	9.27
0.18 veh/m Flooding	54.24%	0.8823	0.8823	0.9980	12.05
0.18 veh/m CPF	75.36%	0.8823	0.8823	0.3567	8.83
0.18 veh/m CPF-NFBB	77.21%	0.8235	0.8339	0.2922	10.05
0.18 veh/m CPF-BPO	76.96%	0.8235	0.8368	0.3058	9.75
0.18 veh/m CPF-BPOD	77.17%	0.8529	0.8529	0.2964	9.53

**Table 5:** Average PDR of three schemes with different *R*.

Vehicle Density	*R* = 300 m			*R* = 500 m			*R* = 800 m		
	CPF	CPF-BPO	CPF-BPOD	CPF	CPF-BPO	CPF-BPOD	CPF	CPF-BPO	CPF-BPOD
0.06 veh/m	79.10%	83.12%	90.30%	83.37%	85.49%	85.80%	89.57%	89.84%	89.77%
0.18 veh/m	66.06%	71.47%	77.89%	75.36%	76.96%	77.17%	81.78%	81.85%	81.68%

## Data Availability

There is no new data created in this study. Data sharing is not available for this paper.

## Abbreviations

The following abbreviations are mainly used in this manuscript:

IoV	Internet of Vehicles
C-V2X	Cellular Vehicle-to-Everything
CBR	Channel Busy Ratio
CSR	Candidate Single-subframe Resources
PDR	Packet Delivery Ratio
PCR	Packet Collision Ratio
PIR	Packet Inter-Reception
MPDR	Multi-hop Packet Delivery Ratio
CPF	CBR-based Probabilistic Forwarding Protocol
CPF-NFBB	CPF Scheme with Non-Forwarding Beyond the Boundary
CPF-BPO	CPF Scheme with Boundary Probability Optimization
CPF-BPOD	CPF Scheme with Boundary Probability Optimization and Delay
veh/m	The number of vehicles per meter
